# Crystal structure and luminescent properties of [1-(biphenyl-4-yl)-1*H*-imidazole-κ*N* ^3^]di­chloridozinc

**DOI:** 10.1107/S2056989015002807

**Published:** 2015-02-13

**Authors:** Xiao-Xiao Liu, Yuan Wang

**Affiliations:** a Changchun University of Science & Technology, School of Chemistry and Environmental Engineering, Changchun 130022, People’s Republic of China

**Keywords:** crystal structure, zinc coordination complex, luminescent properties, π–π inter­actions

## Abstract

The mononuclear title compound, [ZnCl_2_(C_15_H_12_N_2_)_2_], was synthesized by reaction of zinc chloride and 1-(biphenyl-4-yl)-1*H*-imidazole (bpi) under hydro­thermal conditions. The Zn^II^ atom is tetra­hedrally coordinated by the free imidazole N atoms of two bpi ligands and by two Cl atoms. The bpi ligands are not planar, with dihedral angles of 37.52 (14) and 42.45 (14)° between the phenyl rings and 37.13 (14) and 40.05 (14)° between the phenyl rings and the attached imidazole rings, respectively. Mutual π–π inter­actions, with a centroid-to-centroid distance of 3.751 (2) Å between the phenyl and imidazole rings of neighbouring ligands, are present, leading to dimers that are arranged in rows parallel to [-211].

## Abstract 2

A new imidazole-based zinc complex, synthesized using hydro­thermal methods, exhibits luminescent behaviour.

## Chemical context   

Metal coordination polymers constructed from organic ligands and metal cations have received attention because of their structural diversity and inter­esting physical and chemical properties, including adsorption, mol­ecular separation, heterogeneous catalysis and non-linear optics (Sumida *et al.*, 2012 ▸; Colombo *et al.*, 2012 ▸; Henke *et al.*, 2012 ▸). The development of such materials for various applications is reliant on the functionalities and modulations of the inorganic central atoms and the organic linkers. Materials constructed from *d* ^10^ metal ions can be promising photoactive candidates (Lan *et al.*, 2009 ▸; Qin *et al.*, 2014 ▸). For example, a series of zinc- and cadmium-based coordination polymers were reported to be luminescent sensors for the detection of small organic mol­ecules (Yi *et al.*, 2012 ▸; Wang *et al.*, 2013 ▸). On the other hand, the choice of the organic ligands or linkers is important for the supra­molecular arrangement.
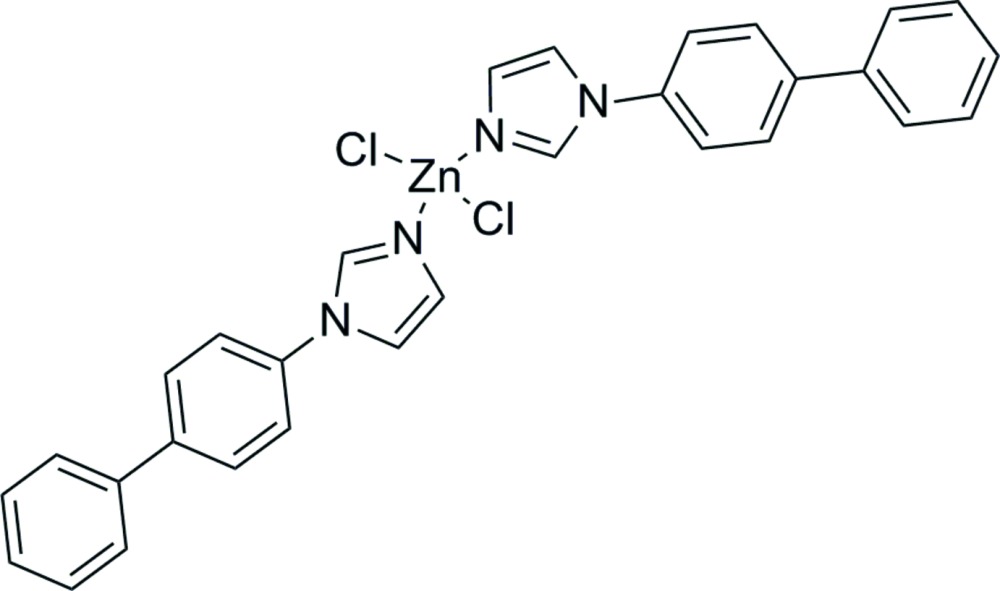


Among the various organic ligands used for the construction of coordination polymers, nitro­gen-donor species are dominant due to their strong affinities for binding metal atoms (Yang *et al.*, 2013 ▸, 2014 ▸). In particular, imidazoles are of great inter­est for the construction of zeolite imidazolate frameworks, which exhibit high stability and practical applications (Phan *et al.*, 2010 ▸). By further modification of imidazole ligands, various compounds with different structural set-ups have been reported, including one-dimensional, two-dimensional and three-dimensional architectures (Kan *et al.*, 2012 ▸). Recently, two one-dimensional imidazole-based zinc complexes were synthesized by using 1,4-di(1*H*-imidazol-1-yl)benzene (dib), and 1,3,5-tri(1*H*-imidazol-1-yl)benzene (tib) as ligands (Wang *et al.*, 2014 ▸). To obtain further effects on the final structure by modification of the substituent of the imidazoles, 1-(biphenyl-4-yl)-1*H*-imidazole (bpi) was chosen as ligand and reacted with Zn^2+^ ions in this work, yielding the title compound ZnCl_2_(C_15_H_12_N_2_)_2_, (I). Apart from the structure determination, its photoluminescent property is also reported.

## Structural commentary   

**Figure 1:**
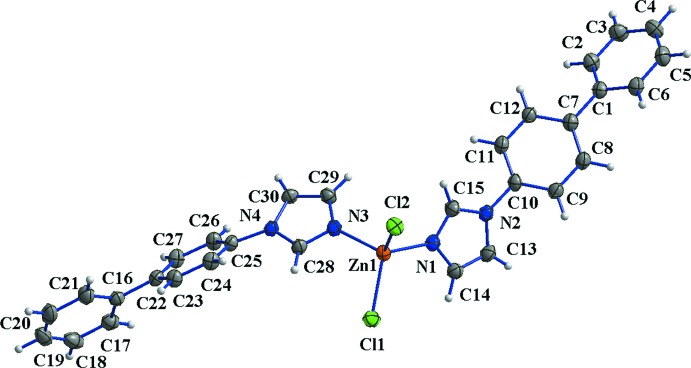
The mol­ecular structure of compound (I). Displacement ellipsoids were drawn at the 30% probability level.

As shown in Fig. 1 ▸, the asymmetric unit of (I) consists of one zinc(II) cation, two bpi ligands and two chlorine ligands. The cation has a distorted tetra­hedral coordination sphere defined by the free imidazole N atoms and two Cl atoms. The Zn—N and Zn—Cl bond lengths (Table 1 ▸) are typical for tetra­hedrally coordinated Zn^II^. The dihedral angles between the two phenyl rings in the two bpi ligands are 37.52 (14) and 42.45 (14)°, respectively, while the dihedral angles between the phenyl rings and the attached imidazole rings are 37.13 (14) and 40.05 (14)°.

**Figure 2:**
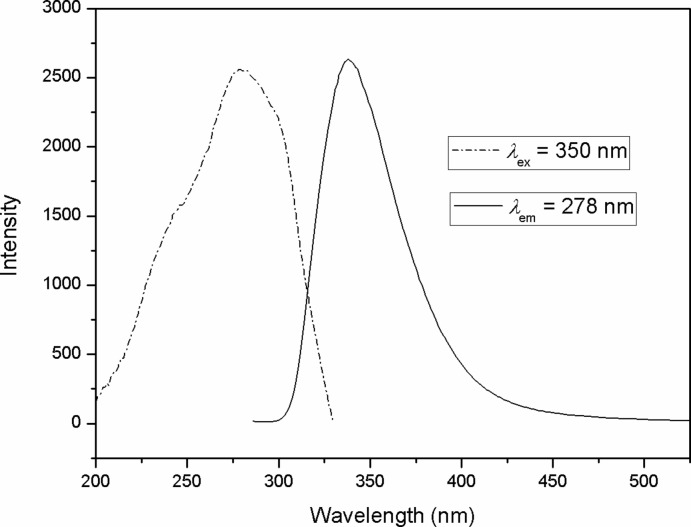
Excitation and emission spectra of compound (I) in the solid state.

Zn^II^-based compounds with metal-organic framework structures are well-known for their luminescence properties. The photoluminescence spectrum of compound (I) in the solid state is shown in Fig. 2 ▸. On excitation at 278 nm, the emission band is centred at 350 nm. Compared to the free bpi ligand, which exhibits one main fluorescent emission band around 400 nm when excited at 271 nm, the emission band of complex (I) is about 50 nm hypochromatically shifted. Considering metal atoms with a *d* ^10^ electron configuration and the bonding inter­actions with the ligand, such broad emission bands may be assigned to a ligand-to-ligand charge transfer (LLCT), admixing with metal-to-ligand (MLCT) and ligand-to-metal (LMCT) charge transfers (Gong *et al.*, 2011 ▸).

## Supra­molecular features   

**Figure 3:**
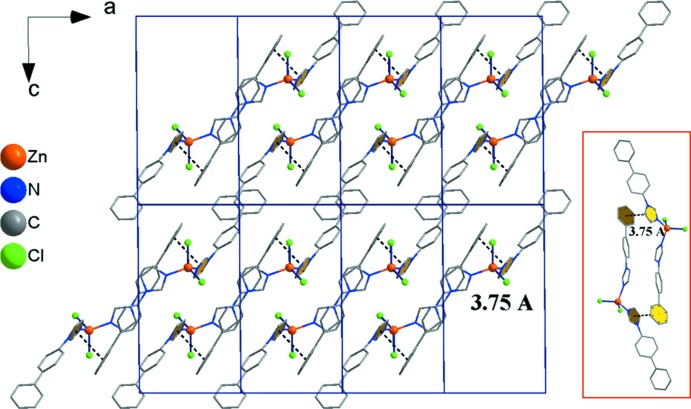
View of the crystal structure along [010] emphasizing π–π inter­actions (dotted lines and inset).

As mentioned before, the imidazole-based ligands dib and tib, featuring two and three imidazole rings, respectively, can adopt different structural dimensionalities. The bpi ligand used in this study, however, has only one available N-donor, thus preventing the formation of a polymeric structure. Nevertheless, there are weak inter­molecular π–π stacking inter­actions between single mol­ecules in the crystal packing. The terminal phenyl ring and the imidazole ring of a neighbouring ligand are tilted to each other by 11.72 (17)°, with a centroid-to-centroid distance of 3.751 (2) Å (Fig. 3 ▸).

## Synthesis and crystallization   

All chemicals were purchased commercially and used without further purification. A mixture of ZnCl_2_ (81.6 mg, 5 mmol), bpi (130 mg, 0.6 mmol), and de-ionized water (9 ml) was loaded into a 20 ml Teflon-lined stainless steel autoclave. The autoclave was sealed and heated at 423 K for 5 d, and then cooled to room temperature by switching off the furnace. Colourless block-shaped crystals were isolated, which were filtered off and washed with de-ionized water. The final product was dried at ambient temperature (yield 75% based on zinc). Analysis calculated (wt%) for ZnCl_2_(C_15_H_12_N_2_)_2_: C, 62.47; H, 4.19; N, 9.71. Found: C, 62.45; H, 4.15; N, 9.79.

Elemental analyses of C, H, and N were conducted on a Perkin–Elmer 2400 elemental analyser. The photoluminescence (PL) excitation and emission spectra were recorded with an F-7000 luminescence spectrometer equipped with a xenon lamp of 450 W as an excitation light source. The photomultiplier tube voltage was 400 V, the scan speed was 1200 nm min^−1^, both the excitation and the emission slit widths were 5.0 nm.

## Refinement   

Crystal data, data collection and structure refinement details are summarized in Table 2 ▸. All hydrogen atoms were positioned geometrically with C—H = 0.93 Å and *U*_iso_(H) = 1.2*U*_eq_(C).

## Supplementary Material

Crystal structure: contains datablock(s) I. DOI: 10.1107/S2056989015002807/wm5118sup1.cif

Structure factors: contains datablock(s) I. DOI: 10.1107/S2056989015002807/wm5118Isup2.hkl

Supporting information file. DOI: 10.1107/S2056989015002807/wm5118Isup3.mol

CCDC reference: 1048515

Additional supporting information:  crystallographic information; 3D view; checkCIF report

## Figures and Tables

**Table 1:** Selected bond lengths ()

Zn1N1	2.021(2)	Zn1Cl1	2.2258(7)
Zn1N3	2.028(2)	Zn1Cl2	2.2447(8)

**Table 2:** Experimental details

Crystal data	
Chemical formula	[ZnCl_2_(C_15_H_12_N_2_)_2_]
*M*_r_	576.80
Crystal system, space group	Triclinic, *P* 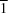
Temperature (K)	296
*a*, *b*, *c* ()	9.2410(6), 9.2595(5), 16.4106(10)
, , ()	87.770(1), 88.819(1), 72.823(1)
*V* (^3^)	1340.50(14)
*Z*	2
Radiation type	Mo *K*
(mm^1^)	1.14
Crystal size (mm)	0.40 0.30 0.30

Data collection	
Diffractometer	Bruker APEXII CCD area detector
Absorption correction	Multi-scan (*SADABS*; Bruker, 2008 ▸)
*T*_min_, *T*_max_	0.658, 0.726
No. of measured, independent and observed [*I* > 2(*I*)] reflections	8564, 5308, 4067
*R*_int_	0.025
(sin /)_max_ (^1^)	0.619

Refinement	
*R*[*F* ^2^ > 2(*F* ^2^)], *wR*(*F* ^2^), *S*	0.037, 0.091, 1.00
No. of reflections	5308
No. of parameters	334
H-atom treatment	H-atom parameters constrained
_max_, _min_ (e ^3^)	0.31, 0.35

Computer programs: *APEX2* and *SAINT* (Bruker, 2008 ▸), *SHELXS97* and *SHELXL97* (Sheldrick, 2008 ▸), *DIAMOND* (Brandenburg, 2006 ▸) and *publCIF* (Westrip, 2010 ▸).

## supplementary crystallographic information

### Crystal data

**Table:**

[ZnCl_2_(C_15_H_12_N_2_)_2_]	*Z* = 2
*M**_r_* = 576.80	*F*(000) = 592
Triclinic, *P*1	#Added by publCIF_symmetry_space_group_name_hall '-P 1' #Added by publCIF_audit_update_record
Hall symbol: -P 1	*D*_x_ = 1.429 Mg m^−^^3^
*a* = 9.2410 (6) Å	Mo *K*α radiation, λ = 0.71073 Å
*b* = 9.2595 (5) Å	Cell parameters from 2594 reflections
*c* = 16.4106 (10) Å	θ = 2.3–24.3°
α = 87.770 (1)°	µ = 1.14 mm^−^^1^
β = 88.819 (1)°	*T* = 296 K
γ = 72.823 (1)°	Block, colourless
*V* = 1340.50 (14) Å^3^	0.40 × 0.30 × 0.30 mm

### Data collection

**Table:**

Bruker APEXII CCD area-detector diffractometer	5308 independent reflections
Radiation source: fine-focus sealed tube	4067 reflections with *I* > 2σ(*I*)
Graphite monochromator	*R*_int_ = 0.025
phi and ω scans	θ_max_ = 26.1°, θ_min_ = 2.3°
Absorption correction: multi-scan (*SADABS*; Bruker, 2008)	*h* = −11→11
*T*_min_ = 0.658, *T*_max_ = 0.726	*k* = −11→11
8564 measured reflections	*l* = −20→17

### Refinement

**Table:**

Refinement on *F*^2^	Primary atom site location: structure-invariant direct methods
Least-squares matrix: full	Secondary atom site location: difference Fourier map
*R*[*F*^2^ > 2σ(*F*^2^)] = 0.037	Hydrogen site location: inferred from neighbouring sites
*wR*(*F*^2^) = 0.091	H-atom parameters constrained
*S* = 1.00	*w* = 1/[σ^2^(*F*_o_^2^) + (0.0389*P*)^2^ + 0.3283*P*] where *P* = (*F*_o_^2^ + 2*F*_c_^2^)/3
5308 reflections	(Δ/σ)_max_ = 0.014
334 parameters	Δρ_max_ = 0.31 e Å^−^^3^
0 restraints	Δρ_min_ = −0.35 e Å^−^^3^

### Special details

**Table:**

Geometry. All e.s.d.'s (except the e.s.d. in the dihedral angle between two l.s. planes) are estimated using the full covariance matrix. The cell e.s.d.'s are taken into account individually in the estimation of e.s.d.'s in distances, angles and torsion angles; correlations between e.s.d.'s in cell parameters are only used when they are defined by crystal symmetry. An approximate (isotropic) treatment of cell e.s.d.'s is used for estimating e.s.d.'s involving l.s. planes.
Refinement. Refinement of *F*^2^ against ALL reflections. The weighted *R*-factor *wR* and goodness of fit *S* are based on *F*^2^, conventional *R*-factors *R* are based on *F*, with *F* set to zero for negative *F*^2^. The threshold expression of *F*^2^ > σ(*F*^2^) is used only for calculating *R*-factors(gt) *etc*. and is not relevant to the choice of reflections for refinement. *R*-factors based on *F*^2^ are statistically about twice as large as those based on *F*, and *R*- factors based on ALL data will be even larger.

### Fractional atomic coordinates and isotropic or equivalent isotropic displacement parameters (Å^2^)

**Table:**

	*x*	*y*	*z*	*U*_iso_*/*U*_eq_	
Zn1	0.49124 (3)	1.09677 (3)	0.333749 (18)	0.04334 (11)	
N3	0.5776 (2)	0.8683 (2)	0.33926 (13)	0.0451 (5)	
N1	0.2746 (2)	1.1586 (2)	0.37469 (13)	0.0472 (5)	
N2	0.0780 (2)	1.2196 (2)	0.45740 (13)	0.0452 (5)	
N4	0.6993 (2)	0.6477 (2)	0.28944 (12)	0.0418 (5)	
C28	0.6686 (3)	0.7984 (3)	0.28125 (16)	0.0466 (6)	
H28A	0.7072	0.8475	0.2395	0.056*	
C25	0.7889 (3)	0.5422 (3)	0.23310 (15)	0.0414 (6)	
C10	−0.0104 (3)	1.2386 (3)	0.53170 (16)	0.0456 (6)	
C7	−0.1776 (3)	1.2747 (3)	0.67615 (16)	0.0452 (6)	
C22	0.9495 (3)	0.3553 (3)	0.11331 (15)	0.0420 (6)	
C30	0.6209 (3)	0.6192 (3)	0.35697 (16)	0.0475 (6)	
H30A	0.6190	0.5249	0.3778	0.057*	
C26	0.7462 (3)	0.4182 (3)	0.21310 (17)	0.0476 (6)	
H26A	0.6641	0.3970	0.2391	0.057*	
C12	−0.0612 (3)	1.1433 (3)	0.66213 (17)	0.0506 (7)	
H12A	−0.0385	1.0662	0.7021	0.061*	
C27	0.8274 (3)	0.3254 (3)	0.15356 (17)	0.0487 (7)	
H27A	0.7994	0.2407	0.1401	0.058*	
C16	1.0323 (3)	0.2598 (3)	0.04652 (16)	0.0453 (6)	
C24	0.9140 (3)	0.5714 (3)	0.19650 (16)	0.0483 (7)	
H24A	0.9449	0.6530	0.2122	0.058*	
C23	0.9918 (3)	0.4791 (3)	0.13708 (17)	0.0477 (6)	
H23A	1.0750	0.4999	0.1120	0.057*	
C6	−0.3160 (3)	1.4297 (3)	0.79101 (18)	0.0519 (7)	
H6A	−0.2935	1.5135	0.7674	0.062*	
C29	0.5470 (3)	0.7554 (3)	0.38738 (16)	0.0492 (6)	
H29A	0.4851	0.7704	0.4336	0.059*	
C11	0.0216 (3)	1.1242 (3)	0.59019 (17)	0.0523 (7)	
H11A	0.0983	1.0349	0.5816	0.063*	
C1	−0.2672 (3)	1.2918 (3)	0.75330 (16)	0.0447 (6)	
C14	0.1475 (3)	1.2263 (3)	0.33028 (18)	0.0524 (7)	
H14A	0.1455	1.2434	0.2740	0.063*	
C8	−0.2076 (3)	1.3875 (3)	0.61520 (17)	0.0517 (7)	
H8A	−0.2861	1.4759	0.6226	0.062*	
C9	−0.1236 (3)	1.3713 (3)	0.54385 (17)	0.0509 (7)	
H9A	−0.1432	1.4493	0.5044	0.061*	
C2	−0.3043 (3)	1.1702 (3)	0.78962 (17)	0.0549 (7)	
H2A	−0.2725	1.0767	0.7655	0.066*	
C17	0.9543 (4)	0.2104 (3)	−0.01281 (18)	0.0578 (7)	
H17A	0.8490	0.2377	−0.0108	0.069*	
C15	0.2278 (3)	1.1556 (3)	0.45106 (17)	0.0511 (7)	
H15A	0.2907	1.1144	0.4948	0.061*	
C5	−0.3974 (3)	1.4436 (3)	0.86282 (19)	0.0605 (8)	
H5A	−0.4282	1.5365	0.8876	0.073*	
C4	−0.4337 (4)	1.3225 (4)	0.89823 (19)	0.0631 (8)	
H4A	−0.4887	1.3327	0.9468	0.076*	
C21	1.1894 (3)	0.2188 (3)	0.0417 (2)	0.0629 (8)	
H21A	1.2441	0.2516	0.0802	0.075*	
C13	0.0260 (3)	1.2647 (3)	0.38006 (17)	0.0550 (7)	
H13A	−0.0737	1.3123	0.3650	0.066*	
C3	−0.3876 (4)	1.1852 (3)	0.86102 (19)	0.0648 (8)	
H3A	−0.4127	1.1025	0.8842	0.078*	
C19	1.1853 (5)	0.0807 (4)	−0.0781 (2)	0.0785 (11)	
H19A	1.2369	0.0203	−0.1197	0.094*	
C18	1.0313 (5)	0.1212 (4)	−0.07452 (19)	0.0727 (10)	
H18A	0.9780	0.0887	−0.1138	0.087*	
C20	1.2645 (4)	0.1285 (4)	−0.0208 (2)	0.0775 (11)	
H20A	1.3697	0.1002	−0.0237	0.093*	
Cl2	0.62413 (8)	1.20582 (8)	0.41117 (4)	0.05347 (18)	
Cl1	0.49053 (9)	1.16111 (8)	0.20162 (4)	0.05715 (19)	

### Atomic displacement parameters (Å^2^)

**Table:**

	*U*^11^	*U*^22^	*U*^33^	*U*^12^	*U*^13^	*U*^23^
Zn1	0.04203 (18)	0.03878 (17)	0.04857 (19)	−0.01084 (13)	0.00502 (13)	−0.00512 (13)
N3	0.0478 (13)	0.0386 (11)	0.0492 (13)	−0.0136 (10)	0.0045 (10)	−0.0034 (10)
N1	0.0405 (12)	0.0491 (13)	0.0514 (14)	−0.0122 (10)	0.0002 (10)	−0.0024 (10)
N2	0.0361 (12)	0.0470 (12)	0.0510 (13)	−0.0098 (10)	0.0007 (10)	−0.0006 (10)
N4	0.0441 (12)	0.0333 (11)	0.0476 (12)	−0.0112 (9)	0.0013 (10)	0.0004 (9)
C28	0.0508 (16)	0.0366 (13)	0.0524 (16)	−0.0137 (12)	0.0069 (13)	0.0018 (12)
C25	0.0402 (14)	0.0333 (13)	0.0488 (15)	−0.0083 (11)	−0.0012 (11)	0.0006 (11)
C10	0.0345 (14)	0.0473 (15)	0.0549 (16)	−0.0118 (12)	0.0042 (12)	−0.0041 (12)
C7	0.0384 (14)	0.0457 (15)	0.0540 (16)	−0.0163 (12)	0.0006 (12)	−0.0039 (12)
C22	0.0388 (14)	0.0367 (13)	0.0499 (15)	−0.0105 (11)	−0.0033 (12)	0.0017 (11)
C30	0.0550 (16)	0.0392 (14)	0.0508 (16)	−0.0186 (13)	0.0026 (13)	0.0049 (12)
C26	0.0426 (15)	0.0400 (14)	0.0636 (18)	−0.0178 (12)	0.0088 (13)	−0.0035 (12)
C12	0.0483 (16)	0.0429 (15)	0.0568 (17)	−0.0083 (13)	0.0007 (13)	0.0026 (12)
C27	0.0476 (16)	0.0387 (14)	0.0646 (18)	−0.0195 (12)	0.0011 (13)	−0.0077 (12)
C16	0.0488 (16)	0.0374 (13)	0.0482 (15)	−0.0112 (12)	0.0033 (12)	0.0020 (11)
C24	0.0478 (16)	0.0431 (14)	0.0598 (17)	−0.0223 (13)	−0.0003 (13)	−0.0035 (13)
C23	0.0400 (14)	0.0460 (15)	0.0603 (17)	−0.0182 (12)	0.0048 (13)	−0.0004 (13)
C6	0.0478 (16)	0.0455 (15)	0.0633 (18)	−0.0151 (13)	0.0034 (14)	−0.0039 (13)
C29	0.0498 (16)	0.0513 (16)	0.0484 (16)	−0.0184 (13)	0.0058 (12)	−0.0016 (12)
C11	0.0436 (16)	0.0441 (15)	0.0628 (18)	−0.0031 (13)	0.0034 (13)	−0.0052 (13)
C1	0.0380 (14)	0.0466 (15)	0.0510 (16)	−0.0146 (12)	−0.0003 (12)	−0.0032 (12)
C14	0.0519 (17)	0.0507 (16)	0.0526 (16)	−0.0129 (14)	−0.0004 (14)	0.0039 (13)
C8	0.0433 (15)	0.0426 (15)	0.0662 (18)	−0.0085 (12)	0.0074 (14)	−0.0018 (13)
C9	0.0437 (15)	0.0459 (15)	0.0603 (18)	−0.0102 (13)	0.0029 (13)	0.0061 (13)
C2	0.0579 (18)	0.0512 (16)	0.0600 (18)	−0.0225 (14)	0.0058 (14)	−0.0088 (14)
C17	0.0657 (19)	0.0519 (17)	0.0575 (18)	−0.0206 (15)	−0.0001 (15)	0.0018 (14)
C15	0.0357 (14)	0.0621 (17)	0.0521 (17)	−0.0091 (13)	−0.0019 (12)	−0.0029 (13)
C5	0.0566 (18)	0.0575 (18)	0.067 (2)	−0.0145 (15)	0.0041 (15)	−0.0157 (15)
C4	0.065 (2)	0.072 (2)	0.0553 (18)	−0.0240 (17)	0.0111 (15)	−0.0064 (16)
C21	0.0498 (18)	0.0642 (19)	0.070 (2)	−0.0103 (15)	0.0076 (15)	0.0011 (16)
C13	0.0379 (15)	0.0604 (18)	0.0594 (18)	−0.0042 (13)	−0.0067 (13)	0.0069 (14)
C3	0.072 (2)	0.0609 (19)	0.066 (2)	−0.0288 (17)	0.0101 (17)	0.0021 (15)
C19	0.116 (3)	0.0500 (19)	0.064 (2)	−0.019 (2)	0.038 (2)	−0.0033 (16)
C18	0.112 (3)	0.0595 (19)	0.0515 (19)	−0.033 (2)	0.0074 (19)	−0.0057 (15)
C20	0.066 (2)	0.061 (2)	0.092 (3)	−0.0024 (18)	0.033 (2)	0.0075 (19)
Cl2	0.0559 (4)	0.0533 (4)	0.0553 (4)	−0.0217 (3)	−0.0010 (3)	−0.0073 (3)
Cl1	0.0716 (5)	0.0505 (4)	0.0496 (4)	−0.0188 (4)	0.0034 (3)	0.0002 (3)

### Geometric parameters (Å, º)

**Table:**

Zn1—N1	2.021 (2)	C16—C17	1.391 (4)
Zn1—N3	2.028 (2)	C24—C23	1.368 (3)
Zn1—Cl1	2.2258 (7)	C24—H24A	0.9300
Zn1—Cl2	2.2447 (8)	C23—H23A	0.9300
N3—C28	1.314 (3)	C6—C5	1.374 (4)
N3—C29	1.377 (3)	C6—C1	1.388 (4)
N1—C15	1.319 (3)	C6—H6A	0.9300
N1—C14	1.367 (3)	C29—H29A	0.9300
N2—C15	1.339 (3)	C11—H11A	0.9300
N2—C13	1.372 (3)	C1—C2	1.381 (4)
N2—C10	1.441 (3)	C14—C13	1.343 (4)
N4—C28	1.341 (3)	C14—H14A	0.9300
N4—C30	1.371 (3)	C8—C9	1.380 (4)
N4—C25	1.434 (3)	C8—H8A	0.9300
C28—H28A	0.9300	C9—H9A	0.9300
C25—C26	1.373 (3)	C2—C3	1.377 (4)
C25—C24	1.384 (3)	C2—H2A	0.9300
C10—C11	1.370 (4)	C17—C18	1.379 (4)
C10—C9	1.376 (3)	C17—H17A	0.9300
C7—C12	1.389 (3)	C15—H15A	0.9300
C7—C8	1.388 (4)	C5—C4	1.367 (4)
C7—C1	1.486 (3)	C5—H5A	0.9300
C22—C27	1.388 (3)	C4—C3	1.379 (4)
C22—C23	1.388 (3)	C4—H4A	0.9300
C22—C16	1.486 (3)	C21—C20	1.388 (4)
C30—C29	1.353 (4)	C21—H21A	0.9300
C30—H30A	0.9300	C13—H13A	0.9300
C26—C27	1.382 (3)	C3—H3A	0.9300
C26—H26A	0.9300	C19—C18	1.361 (5)
C12—C11	1.382 (4)	C19—C20	1.366 (5)
C12—H12A	0.9300	C19—H19A	0.9300
C27—H27A	0.9300	C18—H18A	0.9300
C16—C21	1.390 (4)	C20—H20A	0.9300
			
N1—Zn1—N3	110.09 (9)	C5—C6—C1	120.7 (3)
N1—Zn1—Cl1	108.12 (7)	C5—C6—H6A	119.7
N3—Zn1—Cl1	105.05 (6)	C1—C6—H6A	119.7
N1—Zn1—Cl2	107.94 (7)	C30—C29—N3	109.4 (2)
N3—Zn1—Cl2	111.23 (7)	C30—C29—H29A	125.3
Cl1—Zn1—Cl2	114.33 (3)	N3—C29—H29A	125.3
C28—N3—C29	105.4 (2)	C10—C11—C12	119.2 (2)
C28—N3—Zn1	120.15 (17)	C10—C11—H11A	120.4
C29—N3—Zn1	133.74 (17)	C12—C11—H11A	120.4
C15—N1—C14	105.6 (2)	C2—C1—C6	118.0 (2)
C15—N1—Zn1	127.00 (18)	C2—C1—C7	120.7 (2)
C14—N1—Zn1	127.06 (19)	C6—C1—C7	121.3 (2)
C15—N2—C13	106.9 (2)	C13—C14—N1	109.8 (2)
C15—N2—C10	126.2 (2)	C13—C14—H14A	125.1
C13—N2—C10	126.9 (2)	N1—C14—H14A	125.1
C28—N4—C30	106.8 (2)	C9—C8—C7	121.5 (2)
C28—N4—C25	124.6 (2)	C9—C8—H8A	119.3
C30—N4—C25	128.4 (2)	C7—C8—H8A	119.3
N3—C28—N4	111.9 (2)	C10—C9—C8	119.3 (3)
N3—C28—H28A	124.0	C10—C9—H9A	120.3
N4—C28—H28A	124.0	C8—C9—H9A	120.3
C26—C25—C24	120.8 (2)	C3—C2—C1	121.1 (3)
C26—C25—N4	120.1 (2)	C3—C2—H2A	119.5
C24—C25—N4	119.0 (2)	C1—C2—H2A	119.5
C11—C10—C9	120.8 (2)	C18—C17—C16	120.7 (3)
C11—C10—N2	119.1 (2)	C18—C17—H17A	119.6
C9—C10—N2	120.0 (2)	C16—C17—H17A	119.6
C12—C7—C8	117.5 (2)	N1—C15—N2	111.3 (2)
C12—C7—C1	120.8 (2)	N1—C15—H15A	124.4
C8—C7—C1	121.7 (2)	N2—C15—H15A	124.4
C27—C22—C23	117.6 (2)	C4—C5—C6	120.8 (3)
C27—C22—C16	121.7 (2)	C4—C5—H5A	119.6
C23—C22—C16	120.6 (2)	C6—C5—H5A	119.6
C29—C30—N4	106.4 (2)	C5—C4—C3	119.2 (3)
C29—C30—H30A	126.8	C5—C4—H4A	120.4
N4—C30—H30A	126.8	C3—C4—H4A	120.4
C25—C26—C27	118.8 (2)	C16—C21—C20	119.7 (3)
C25—C26—H26A	120.6	C16—C21—H21A	120.2
C27—C26—H26A	120.6	C20—C21—H21A	120.2
C11—C12—C7	121.6 (3)	C14—C13—N2	106.4 (2)
C11—C12—H12A	119.2	C14—C13—H13A	126.8
C7—C12—H12A	119.2	N2—C13—H13A	126.8
C26—C27—C22	121.7 (2)	C2—C3—C4	120.2 (3)
C26—C27—H27A	119.1	C2—C3—H3A	119.9
C22—C27—H27A	119.1	C4—C3—H3A	119.9
C21—C16—C17	118.5 (3)	C18—C19—C20	120.3 (3)
C21—C16—C22	120.6 (3)	C18—C19—H19A	119.9
C17—C16—C22	120.8 (2)	C20—C19—H19A	119.9
C23—C24—C25	119.4 (2)	C19—C18—C17	120.1 (3)
C23—C24—H24A	120.3	C19—C18—H18A	119.9
C25—C24—H24A	120.3	C17—C18—H18A	119.9
C24—C23—C22	121.5 (2)	C19—C20—C21	120.7 (3)
C24—C23—H23A	119.2	C19—C20—H20A	119.7
C22—C23—H23A	119.2	C21—C20—H20A	119.7
